# Healthcare Workers after Two Years of COVID-19: The Consequences of the Pandemic on Psychological Health and Sleep among Nurses and Physicians

**DOI:** 10.3390/ijerph20021410

**Published:** 2023-01-12

**Authors:** Valentina Alfonsi, Serena Scarpelli, Maurizio Gorgoni, Alessandro Couyoumdjian, Francesco Rosiello, Cinzia Sandroni, Roberto Corsi, Filomena Pietrantonio, Luigi De Gennaro

**Affiliations:** 1 Department of Psychology, Sapienza University of Rome, 00185 Rome, Italy; 2 IRCCS Fondazione Santa Lucia, 00179 Rome, Italy; 3 Internal Medicine Department, Ospedale dei Castelli, Azienda Sanitaria Locale Roma 6, Ariccia, 00040 Rome, Italy; 4 UOC Professione Infermieristica, Azienda Sanitaria Locale Roma 6, Albano, 00041 Rome, Italy; 5 Direzione Sanitaria Aziendale, Azienda Sanitaria Locale Roma 6, Albano, 00041 Rome, Italy; 6 Department of Health Economics, St. Camillus International University of Health Sciences, 00131 Rome, Italy

**Keywords:** COVID-19 outbreak, nurses, physicians, healthcare workers, traumatic event, sleep quality, stress, depression, anxiety

## Abstract

COVID-19 has challenged the health workforce worldwide. In this cross-sectional study with a retrospective assessment, we explored the impact of the pandemic on mental health and sleep among a sample of Italian nurses and medical doctors. A total of 287 healthcare workers (212 nurses and 75 physicians) completed a web survey on socio-demographic, psychological, and sleep-related aspects referring to the period before the pandemic and to the present period of February to June 2022. Comparisons between nurses and physicians revealed that the former had greater distress in response to the pandemic. Consistently, the multivariate analysis of covariance showed that even if both groups were negatively impacted by the pandemic, nurses presented a greater worsening over time regarding several psychological and sleep symptoms. Furthermore, we observed that working on the frontline represented an additional risk factor for nurses. In line with previous evidence, we also found that personal experiences with COVID-19 are significant predictors of the current health status. Our results underscore the urgent need for preventive programs among healthcare operators to increase their coping skills and prevent the long-term consequences of chronic stress, especially for high-risk professionals. Specific attention should also be devoted to programs to improve sleep quality and reduce sleep-related traumatic symptoms.

## 1. Introduction

The coronavirus disease (COVID-19) pandemic represents the biggest global health crisis of our time [1,2]. Unlike other workers who reduced or did not modify their workplace settings, medical and paramedical staff had to intensify their professional activity during the acute phases of the pandemic [3].

Several studies documented the detrimental consequences on both physical and mental health in this population [4,5]. In Italy, from the beginning of the pandemic, more than 260,000 COVID-19 cases were recorded among healthcare workers (HCWs) (~2.5% of the general population) [6]. 

The heightened vulnerability among HCWs has been associated with several factors. First of all, the greater likelihood of COVID-19 infection places this professional category in a particularly disadvantaged position [7,8]. Further, the most frequent virus transmission among households and colleagues may represent an additional psychosocial burden [9]. We should also consider that constant exposure to an emergency context increases the risk for occupational stress and burnout [10], which in turn leads to the onset of physical or mental disease [11]. Concerns over their responsibility and the pressure of social expectations also weigh on HCWs’ professional experiences [12]. 

Not unexpectedly, working on the frontline within this unprecedented scenario represents the leading risk factor for the onset of psychological disorders [13,14]. On the other hand, older age and more work experience would seem to be protective elements associated with greater management skills [15,16]. 

All of these work-related factors added to individual experiences with COVID-19 (e.g., getting infected with COVID-19, quarantine period, relatives or friends who died from COVID-19, etc.), which were found to be significant determinants of the negative impact of COVID-19 on the general population [17,18].

A plethora of evidence showed the alarming deterioration of the well-being of HCWs during the pandemic. In particular, HCWs reported feeling higher levels of anxiety, stress, and depression than before the pandemic [19], along with a significant sleep quality impairment [20]. Moreover, as the COVID-19 outbreak is comparable to a real traumatic event—especially regarding this population—higher post-traumatic stress disorder (PTSD) symptoms were also documented among healthcare staff [21]. It should also be considered that, during the pandemic, a relevant phenomenon of opposition by health professionals to national health directives occurred. A quite large number of HCWs joined the NoVax, AntiVax, and Freevax movements (i.e., ~0.70% in Italy and 1.92% in our sample), which denied the usefulness of vaccines and opposed the pandemic management methods. These HCWs were, in a second phase, removed from work, which added a further burden for the remaining healthcare personnel [22,23].

In most of the studies, the two most representative categories of health professionals were examined: nurses and physicians. Although converging evidence indicates a worse health status for nurses than for medical doctors, only a few studies have directly compared the outcomes of the pandemic between these two groups [24]. In this regard, none investigated the respective overtime changes from the period before the COVID-19 outbreak to the current advanced stage of the pandemic in Italy (i.e., the end of the state of emergency was declared in Italy on 31 March 2022).

In light of the previous literature about the detrimental effects of the pandemic on HCWs across different COVID-19 waves, the main purpose of the present study was to investigate the current mental health status and sleep quality relative to the pre-pandemic within a large population of Italian HCWs, focusing on the potential differences between medical doctors and nurses.

Furthermore, given the crucial relevance of working on the frontline (i.e., working directly with COVID-19-positive patients) [25], we also assessed its role separately for medical doctors and nurses. 

Finally, we investigated the predictive value of the subjective experience with COVID-19 in determining the current health status and sleep quality among the entire sample of HCWs.

We expect to confirm the worsening effect of the pandemic, even beyond its acute phase, with a greater impact on the category of nurses than medical doctors. Consistently, we expect that working on the frontline still represents a risk factor, especially for these types of healthcare professionals. Finally, in line with the evidence on the general population, we hypothesize an explanatory role of the close experience with COVID-19 in determining the negative outcome of the pandemic on sleep and mental health in HCWs.

## 2. Materials and Methods

### 2.1. Participants and Protocol

We conducted a cross-sectional study with a retrospective assessment. The data collection was carried out through a web-based survey implemented with Google Forms. The survey took approximately 20 min to complete and was enabled from February 2022 to June 2022. The study was reported according to the Checklist for Reporting Results of Internet E-Surveys (CHERRIES) (Supplementary Materials S1). 

All HCWs of the Azienda Sanitaria Locale (ASL) Roma 6 were invited via mail to participate. 

The Local Health Authority “Azienda ASL Roma 6” is a Public Health Authority in the Lazio Region in Central Italy. It is staffed with 3312 employees (including administration and healthcare providers). It includes 4 hospital structures, 8 medium-intensity private structures, and at least 36 low-intensity territorial facilities for vulnerable patients.

After a brief section to assess socio-demographic, occupational, and COVID-19-related data, participants were requested to fill out some questionnaires to assess sleep and psychological aspects considering both the pre-pandemic and current periods (Supplementary Materials S2).

All individuals completed the survey after reading the informed consent and declaring their explicit agreement to participate in the research. Participants could withdraw from the study at any time without any justification and no data was saved. No personally identifiable information was collected to guarantee anonymity. Participation was voluntary and without monetary compensation. 

All study procedures were conducted in accordance with the Declaration of Helsinki and were approved by the local ethics committee (Comitato Etico “LAZIO 2”, prot. N. 0223816/2021).

### 2.2. Measures

- Socio-demographic, occupational and COVID-19-related information: A short section first collected socio-demographic information (i.e., age, gender, marital status, education level). Then, some questions investigated specific information about the healthcare career (i.e., healthcare profession, department, length of service, working on the frontline). Finally, we collected COVID-19-related information (i.e., COVID-19 positivity, forced quarantine period, COVID-19-infected relatives or friends, relatives or friends who died from COVID-19, satisfaction with governmental measures).
- Impact of Event Scale (IES) [26]: An Italian validation of a self-administered questionnaire to assess the symptoms of PTSD after previous traumatic event experiences. This 15-item scale is composed of different emotional reactions to which the respondent is asked to indicate how frequently each reaction was experienced from 0 to 4, where 0 indicates “Not at all” and 4 indicates “Extremely”.
- Pittsburgh Sleep Quality Index (PSQI) [27]: An Italian validation of a self-reported questionnaire to investigate subjective sleep quality. The measure consists of 19 items, resulting in partial scores in 7 subscales (subjective sleep quality, sleep latency, sleep duration, habitual sleep efficiency, sleep disturbances, use of sleep medications, daytime dysfunction) and a global score. A PSQI global score > 5 indicates poor subjective sleep quality.
- Pittsburgh Sleep Quality Index-Addendum (PSQI-A) [28]: A specific self-report measure for the assessment of seven disruptive nocturnal behaviors common among subjects with PTSD: flashes, general nervousness, memories or nightmares of traumatic experience, severe anxiety or panic not related to traumatic memories, bad dreams not related to traumatic memories, episodes of terror or screaming during sleep without fully awakening, and episodes of acting out dreams, such as kicking, punching, running, or screaming. A PSQI score > 4 is highly predictive for discriminating between subjects with and without PTSD.
- Depression Anxiety Stress Scale (DASS-21) short form [29]: An Italian validation of a self-report questionnaire in which participants rate the frequency and severity of depression, anxiety, and stress symptoms. The 21 Items consist of sentences about the previous week and each item is scored on a 4-point Likert scale, ranging from 0 (“I strongly disagree”) to 3 (“I totally agree”). Subscale scores are calculated as the sum of scores for the relevant seven items from each subscale. The cut-offs are obtained by multiplying the raw scores by two to suit the original 42 items and the scores indicative of severe rating of depression, anxiety, and stress are ≥21, ≥15, and ≥26, respectively.

### 2.3. Statistical Analyses 

Descriptive statistics were conducted to outline the socio-demographic, occupational, and COVID-19-related characteristics in the whole sample and in the two categories of healthcare providers (nurses and physicians). 

To estimate the effects of the pandemic on the impact of the traumatic event (IES total score) between the two different groups of HCWs, we first conducted a two-way analysis of covariance (ANCOVA) considering “Profession” and “Gender” as between-subjects factors, and “Years on the job” as a covariate. 

Then, considering sleep- (PSQI total score, PSQI-A score) and psychological- (DASS-Depression, DASS-Anxiety, and DASS-Stress scores) dependent variables, a two-way mixed multivariate ANCOVA (MANCOVA) was carried out considering “Time” (pre-pandemic period: “T0” vs. current period: “T1”) and “Profession” (nurses vs. physicians) as within- and between-subjects factors, respectively, and “Years on the job” as a covariate. 

Secondly, with the aim to explore the impact of the current frontline engagement on sleep and psychological characteristics across the two time points in both nurses and physicians, we conducted two separates mixed MANCOVAs with “Time” (pre-pandemic period: “T0” vs. current period: “T1”) and “Working frontline” (Yes vs. No) as within- and between-subjects factors, respectively, and “Years on the job” as a covariate. 

Finally, multiple linear regressions were performed to assess the best COVID-19-related predictors (i.e., COVID-19 positivity, forced quarantine period, COVID-19-infected relatives or friends, relatives or friends who died from COVID-19, satisfaction with governmental measures) for the current scores on sleep and psychological well-being in the whole sample. Collinearity was checked using a normal linear regression collinearity diagnostic test. No variance inflation factor ≥5 was observed. 

Before running the above tests, the assumption of normality was checked. For each analysis, *p*-values ≤ 0.05 were considered statistically significant. 

The statistical analyses were performed using the Statistical Package for Social Sciences (SPSS) version 25.0 and Matlab R2016.

## 3. Results

### 3.1. Characteristics of the Sample and Their Distributions 

A total of 287 HCWs (212 nurses and 75 physicians) completed the survey. The characteristics of the participants (total sample, nurses, physicians) are shown in Table 1. The participants’ mean age was 46.12 (SE ± 0.66) and the average length of service was 18.45 years (SE ± 0.72). Most of the participants were females (76.4%). Among all respondents, 35.9% were single, 47.0% were married or cohabiting, and a small percentage were divorced/separated/widower (16.4%). Most of the individuals received higher education (97.6%). Concerning occupational characteristics, 37.6% reported working on the frontline at the time of the survey. A total of 123 participants (42.9%) had COVID-19 during the pandemic and a forced quarantine period was prescribed to 148 (51.9%) individuals. Most of the respondents had COVID-19-infected relatives or friends (72.5%) and 14.3% had relatives or friends who had died from COVID-19. Finally, 204 HCWs declared their agreement with the restrictive measures adopted by the government. 

The comparison between the two subgroups of HCWs mirrored some intrinsic differences between categories, such as the proportion of female workers among nurses, the length of service and the educational levels. The demographic data collected on our sample are roughly consistent with the demographics of Italian HCWs in general (e.g., [6,30]).

### 3.2. The Effects of the Pandemic on Nurses and Physicians

**Figure 1:**
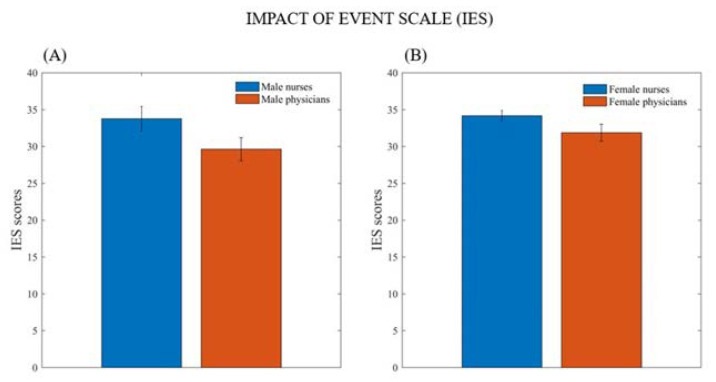
Mean (and SE) of Impact of Event Scale (IES) total score in male nurses (n = 45) and physicians (n = 28) and (**A**) Mean (and SE) of Impact of Event Scale (IES) total score in female nurses (n = 167) and physicians (n = 47) (**B**).

The two-way ANCOVA showed that both male and female nurses exhibited higher impacts of traumatic events than male and female medical doctors (F_1,282_ = 7.425, *p* = 0.007) (Figure 1). In addition, the length of service acted as a protective factor (F_1,282_ = 10.590, *p* = 0.001).

The two-way mixed MANCOVA (“Time” vs. “Profession”; covariate: “Years on the job”) performed on sleep and psychological variables showed statistically significant differences between the two groups (Pillai’s Trace = 0.109, F_5,280_ = 6.824, *p* < 0.001) and the two time points (Pillai’s Trace = 0.317, F_5,280_ = 25.993, *p* < 0.001) and significant interaction between these main effects (Pillai’s Trace = 0.042, F_5,280_ = 2.448, *p* = 0.034) after controlling for the covariate “Years on the job” (Pillai’s Trace = 0.050, F_5,280_ = 2.926, *p* = 0.014).

**Figure 2:**
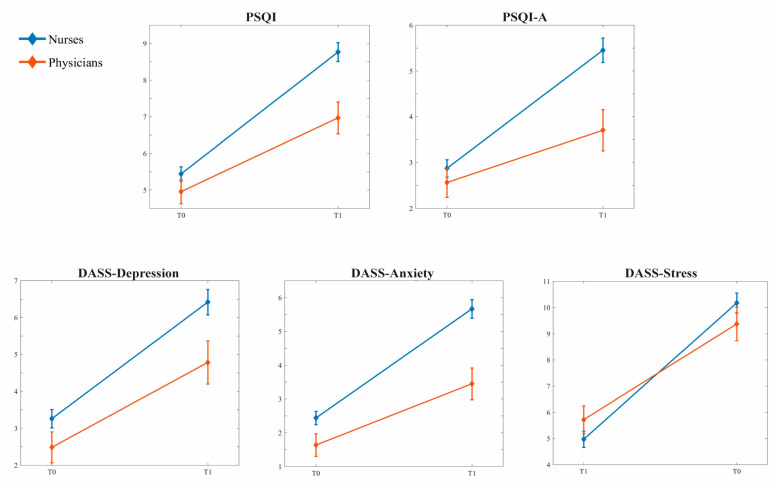
Mean (and SE) across the two time periods (T0: before the pandemic; T1: current period) in the two groups of HCWs (212 nurses and 75 physicians) for the following variables: total scores of Pittsburgh Sleep Quality Index (PSQI), total scores Pittsburgh Sleep Quality Index-Addendum (PSQI-A), scores on each scale of Depression Anxiety Stress Scale (DASS-21).

Specifically, subsequent ANOVAs on each dependent variable (Table 2 and Figure 2) revealed that—independently from the time interval—nurses showed worse conditions on all examined dimensions (PSQI, PSQI-A, DASS-Depression, DASS-Anxiety), except for DASS-Stress. In addition, both groups reported higher scores (i.e., worsening) on all sleep and psychological scales during the current phase than before the pandemic. However, the “Time” vs. “Profession” interaction points to a greater pandemic-related worsening in nurses compared with physicians on PSQI, PSQI-A, DASS-Anxiety, and DASS-Stress scores. 

### 3.3. The Impact of Frontline Engagement on Nurses and Physicians

The two-way mixed MANCOVAs (“Time” vs. “Working frontline”; covariate: “Years on the job”) for nurses and medical doctors on sleep and psychological variables showed different results. In both groups, the main effect of “Time” was statistically significant (Pillai’s Trace = 0.382, F_5,205_ = 25.368, *p* < 0.001 [nurses]; Pillai’s Trace = 0.280, F_5,68_ = 5.280, *p* < 0.001 [physicians]), whereas the main effect of “Working frontline” did not reach significance. However, the interaction between “Time” and “Working frontline” was significant only for the nursing staff (Pillai’s Trace = 0.063, F_5,205_ = 2.764, *p* = 0.019 [nurses]; Pillai’s Trace = 0.035, F_5,68_ = 0.487, *p* = 0.785 [physicians]) after controlling for the covariate “Years on the job” (Pillai’s Trace = 0.046, F_5,205_ = 1.963, *p* = 0.086 [nurses]; Pillai’s Trace = 0.162, F_5,68_ = 2.634, *p* = 0.031 [physicians]). 

**Figure 3:**
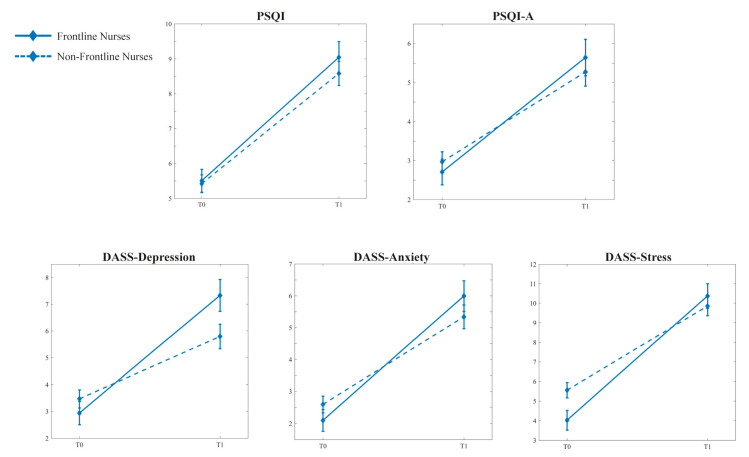
Mean (and SE) across the two time periods (T0: before the pandemic; T1: current period) among frontline (n = 80) and non-frontline (n = 132) nurses working for the following variables: total scores of Pittsburgh Sleep Quality Index (PSQI), total scores Pittsburgh Sleep Quality Index-Addendum (PSQI-A), scores on each scale of Depression Anxiety Stress Scale (DASS-21).

In this subgroup, subsequent ANOVAs on each dependent variable (Table 3) revealed that nurses working on the frontline exhibited a more marked worsening following the pandemic on psychological variables (DASS-Depression, DASS-Anxiety, and DASS-Stress) than nursing not working on the frontline (Figure 3). 

### 3.4. COVID-19-Related Predictors of the Current Sleep and Psychological Conditions in Healthcare Workers

Results of the multiple linear regressions on the PSQI, PSQI-A, DASS-Depression, DASS-Anxiety, and DASS-Stress scores considering COVID-19-related personal experiences as predictors are depicted in Table 4. The multiple regression coefficients were statistically significant for each sleep and psychological variable.

The partial correlations indicate that higher scores on sleep and psychological scales are consistently correlated with having friends or relatives who have died from COVID-19 (positive correlation) and with the degree of agreement on the restrictive measures established by the Italian government (negative correlation). 

## 4. Discussion

To the best of our knowledge, this is the first Italian study aimed at retrospectively exploring the overtime changes from the period before COVID-19 to the current stage of the pandemic separately for nurses and medical doctors. 

Our findings confirmed and extended the previous literature, indicating the negative effects of the pandemic on psychological- and sleep-related aspects among healthcare professionals and especially among nursing staff [24], for whom working on the frontline represented an additional risk factor. In line with previous literature [17,18,31], we also identified personal experiences with COVID-19-related events (i.e., relatives or friends who have died from COVID-19) and the degree of agreement with the restrictive measures imposed by the government as significant predictors of the current health status among nurses. 

As expected, the pandemic’s negative impact concurrently affected various mental well-being domains.

Available evidence has demonstrated increased adverse psychological outcomes among HCWs during the pandemic [4,32]. Accordingly, we observed more severe depression, anxiety, and stress symptoms over the two-year period. We also found that HCWs decreased their self-perceived sleep quality during the current phase of the COVID-19 outbreak, as reflected by higher scores than they reported remembering experiencing in the pre-pandemic period. Many studies have described the high rates of insomnia and sleep problems among HCWs during the pandemic, and a recent systematic review and meta-analysis found a pooled prevalence of 42% in this population [5]. In addition to the well-described factors determining the COVID-Somnia symptoms in the general population (e.g., reduced impact of social zeitgebers, increase of digital media use near bedtime, psychological distress, etc.) [33,34], HCWs were also exposed to risk factors closely related to their profession. Namely, the strenuous work shifts during the pandemic (unusual, long, consecutive), feelings of burnout, and the higher odds of COVID-19 infection may explain the greater prevalence of sleep disturbances among healthcare professionals than in the general population (42% vs. 18–31%) [5].

Remarkably, sleep dysfunction negatively affects the subsequent diurnal performance, compromising the quality of care and increasing the risk of workplace accidents [35,36]. 

Alongside the adverse consequences on sleep quality, we also found increased sleep-related traumatic symptoms following the pandemic. As shown by several studies, the effects of the COVID-19 outbreak were comparable to those of a traumatic event [37]. Thus, the appearance of nocturnal PTSD symptoms, such as increased nightmare frequency, may represent a natural sequela and a sort of attempt to cope with the negative emotions arising from emergency condition [38]. 

The significant effect of the covariate “Years on the job” is in keeping with prior research studies on the role of the length of service, which demonstrated a protective function thanks to a better ability to adapt to emergencies [15]. Indeed, we found that work experience mitigates HCWs’ vulnerability to developing psychological symptoms and sleep problems. Moreover, the recruitment of young and inexperienced people (i.e., medical students) to manage the lack of human resources during the pandemic [39] may have further strengthened this finding. 

Starting from the consolidated evidence that different healthcare providers are differently affected by the pandemic [24], we also aimed to investigate the potential variability between nurses and medical doctors. We found worse psychological conditions and sleep quality for nurses than medical doctors in both the pre-pandemic and current periods. This finding is in line with the fact that nursing staff already showed high rates of mental and sleep diseases under normal circumstances, as observed in pre-COVID studies [40,41]. 

Regarding the different impacts of COVID-19 on nurses and physicians, we show that both groups are negatively affected by the pandemic, even though nurses are more vulnerable to its detrimental effects. Specifically, our results point to a greater worsening of anxiety, stress, sleep quality, and sleep-related traumatic symptoms than for the physicians. 

Many studies have documented the higher costs of the pandemic for nurses [42], and multiple contextual and socio-demographic variables were identified as potential explanatory factors for such differences. First of all, nurses experienced an additional workload as they appeared to have offered more extended and direct care (i.e., one-to-one) for COVID-19 patients than medical doctors [39,43]. Furthermore, the reallocation of human resources mainly due to the shortage of personnel may have affected nursing staff more than other categories [44].

Socio-demographic variables may also have triggered adverse outcomes among nurses. Indeed, the current literature indicates that women expressed more detrimental consequences of the pandemic compared to men, probably due to the increased social and family duties [45]. It is well known that the majority of nurses are women, and consequently, the worse conditions among nurses during the pandemic could be partially ascribed to this aspect. With respect to the protective role of the length of service, the younger age of nurses in our sample may represent another risk factor linked to less work experience. Finally, studies also described the lesser experience in crisis management among nurses than physicians [46].

Unfortunately, we did not have information about the specific type of work shift that may have modulated the negative impact of the pandemic [47]. 

To summarize, the COVID-19 pandemic seems to have amplified a pre-existing distress condition among nurses. 

In general, healthcare staff directly involved in the care of COVID-19 patients (i.e., frontline operators) had an increased chance of developing sleep alteration and negative psychological outcomes [20]. In line with the hypothesis of the higher susceptibility of nurses, we found that being a frontline worker represented an additional risk factor for the harmful effects of the pandemic over time, which was larger in the subgroup of nurses. 

The subjective experience of COVID-19 or COVID-related events modulates the severity of the negative outcomes in the general population [17,18,48]. Likewise, our results showed that HCWs that have COVID-related traumatic experiences such as the death of relatives or friends because of the pandemic experienced more psychological distress, more nocturnal PTSD symptoms, and poorer sleep quality. Further, we also found that nurses and medical doctors who had low trust in the restrictive measures adopted by the government to deal with the health emergency reported higher levels of psychological and sleep symptoms.

Taken together, these latest findings suggest that the proximity to the traumatic event and the perceived validity of the government policies are significant determinants of the negative consequences of the pandemic among healthcare staff. Accordingly, a recent scoping review highlighted that the effects of the pandemic on HCWs are closely related to individual, interpersonal, and also institutional aspects [49]. 

## 5. Limitations

The retrospective nature of our survey may have led to an overestimation of the detrimental effects of the pandemic due to a recall bias. Indeed, the ex post facto assessment of pre-pandemic sleep and psychological conditions may have been influenced by the current health status. We should also consider that subjective experiences such as dissatisfaction with the work conditions, salary, appreciation, etc., at the time of completing the survey could have further biased these results. Although this type of study allows for the collection of data on relatively large samples, the lack of prospective measures (e.g., sleep diaries) or objective tools (e.g., polysomnographic or actigraphic recordings) to assess sleep and psychological variables represents an intrinsic limit and restricts the generalizability of the results. In addition, although other surveys found similar results among HCWs from other regions of Italy (e.g., [6]), the fact that we only considered the medical staff of Lazio prevents the results from being generalized to the entire national population of HCWs.

Further, our results may be partially confounded by the socio-demographic differences between the nurses and physicians. However, the younger age and the mostly female gender among nurses mirrored the natural composition of the two groups of HCWs [50], and our study aimed to observe the effects of the pandemic within ecological healthcare settings. Similarly, the numerical prevalence of nurses compared to physicians reflects the effective distribution of personnel within the local health unit. 

Nevertheless, future studies should take certain demographic differences into consideration to estimate their specific contributions to the observed variations. 

## 6. Conclusions

In conclusion, our study described the lingering impact of the pandemic on HCWs, especially on nurses, who represent the largest group of healthcare professionals. Given the essential role of HCWs within the health systems, effective treatment strategies in order to increase their resilience in the contexts of high work pressure (i.e., pandemics) and to manage the long-term implications on physical and mental health are of critical importance. Thus, our results highlight the importance of developing targeted intervention and prevention programs taking into account the risk and protective factors, especially for high-risk professionals. Although some measures have been implemented by the ASL Roma 6 to contain the spread of the COVID-19 infection (i.e., swabs every 20 days, hand washing campaign), specific programs aimed at reducing the short- and long-term consequences of the pandemic on mental and sleep health are still lacking. Future studies should explore the effectiveness of potential countermeasures, such as Psychoeducational programs or sleep hygiene interventions.

## Figures and Tables

**Table 1:** Socio-demographic, occupational, and COVID-19-related characteristics in the total sample, nurses and physicians.

	Total Sample (N = 287)	Nurses (n = 212)	Physicians (n = 75)	Nurses vs. Physicians
	Mean (SE)			t (*p*)
Age	46.12 (0.66)	45.41 (0.75)	48.13 (1.34)	1.829 (0.069)
Years on the job	18.45 (0.72)	19.71 (0.82)	14.87 (1.40)	−3.007 (0.003) *
	N° (%)			χ^2^ (*p*)
Gender				
Male	73 (25.4)	45 (21.2)	28 (37.3)	7.578 (0.006) *
Female	214 (76.4)	167 (78.8)	47 (62.7)	
Marital status				
Single	103 (35.9)	76 (35.8)	27 (36.0)	8.841 (0.031) *
Married/Cohabitant	135 (47.0)	92 (43.4)	43 (57.3)	
Divorced/Separated	47 (16.4)	42 (19.8)	5 (6.7)	
Widower	2 (0.7)	2 (0.9)	0 (0.0)	
Education level				
Until middle School	7 (2.4)	7 (3.3)	0 (0.0)	171.061 (<0.001) **
High School	51 (17.8)	51 (24.1)	0 (0.0)	
Bachelor’s Degree	122 (42.5)	122 (57.5)	0 (0.0)	
Master’s Degree	99 (34.5)	29 (13.7)	70 (93.3)	
PhD/postgraduate school	8 (2.8)	3 (1.4)	5 (6.7)	
Currently working on the frontline				
Yes	108 (37.6)	80 (37.7)	28 (37.3)	0.004 (0.951)
No	179 (62.4)	132 (62.3)	47 (62.7)	
COVID-19 positivity during the pandemic				
Yes	123 (42.9)	86 (40.6)	37 (49.3)	1.739 (0.187)
No	164 (57.1)	126 (59.4)	38 (50.7)	
Forced quarantine period				
Yes	148 (51.6)	113 (53.3)	35 (46.7)	0.977 (0.323)
No	139 (48.4)	99 (46.7)	40 (53.3)	
COVID-19-infected relatives or friends				
Yes	208 (72.5)	152 (71.7)	56 (74.7)	0.245 (0.621)
No	79 (27.5)	60 (28.3)	19 (25.3)	
Relatives or friends who have died of COVID-19				
Yes	41 (14.3)	36 (17.0)	5 (6.7)	4.813 (0.028) *
No	246 (85.7)	176 (83.0)	70 (93.3)	
Satisfaction with governmental measures				
Yes	204 (71.1)	151 (71.2)	53 (70.7)	0.008 (0.927)
No	83 (28.9)	61 (28.8)	22 (29.3)	

Abbreviation: SE, standard error. * Asterisks indicate statistical significance (*p* ≤ 0.05). ** Asterisks indicate statistical significance (*p* < 0.001).

**Table 2:** Univariate test of the two-way mixed MANCOVA with “Years on the job” as covariate and “Time” and “Profession” as within and between factors, respectively.

	Time		Profession		Time * Profession		Covariate (Years on the Job)	
	F_1,284_	*p*	F_1,284_	*p*	F_1,284_	*p*	F_1,284_	*p*
PSQI	61.558	<0.001 **	8.951	0.003 *	7.406	0.007 *	0.002	0.961
PSQI-A	43.389	<0.001 **	6.909	0.009 *	9.096	0.003 *	1.385	0.240
DASS-Depression	58.371	<0.001 **	5.423	0.021 *	2.200	0.139	0.506	0.478
DASS-Anxiety	70.281	<0.001 **	14.018	<0.001 **	8.006	0.005 *	8.429	0.004 *
DASS-Stress	122.702	<0.001 **	0.002	0.966	4.629	0.032 *	4.702	0.031 *

* Asterisks indicate statistical significance (*p* ≤ 0.05). ** Asterisks indicate statistical significance (*p* < 0.001).

**Table 3:** Univariate test of the two-way mixed MANCOVAs with “Years on the job” as covariate and “Time” and “Working frontline” as within and between factors, respectively, on nurses (n = 212) and physicians (n = 75).

	Time		Working Frontline		Time * Working Frontline		Covariate (Years on the Job)	
Nurses								
	F_1,209_	*p*	F_1,209_	*p*	F_1,209_	*p*	F_1,209_	*p*
PSQI	62.036	<0.001 **	0.426	0.515	0.473	0.492	0.010	0.920
PSQI-A	54.284	<0.001 **	0.016	0.900	1.372	0.243	0.620	0.432
DASS-Depression	55.638	<0.001 **	0.737	0.392	11.282	0.001 *	1.103	0.295
DASS-Anxiety	75.426	<0.001 **	0.030	0.863	4.333	0.039 *	5.733	0.018 *
DASS-Stress	122.662	<0.001 **	0.659	0.418	7.311	0.007 *	4.265	0.040 *
Physicians								
	F_1,72_	*p*	F_1,72_	*p*	F_1,72_	*p*	F_1,72_	*p*
PSQI	12.884	0.001 *	0.586	0.446	0.784	0.379	0.090	0.765
PSQI-A	3.113	0.082	0.056	0.814	0.023	0.880	0.881	0.351
DASS-Depression	11.272	0.001 *	4.327	0.041	0.554	0.463	2.391	0.126
DASS-Anxiety	10.900	0.001 *	0.903	0.345	1.944	0.168	1.827	0.181
DASS-Stress	20.850	<0.001 **	0.164	0.687	1.031	0.313	0.749	0.390

* Asterisks indicate statistical significance (*p* ≤ 0.05). ** Asterisks indicate statistical significance (*p* < 0.001).

**Table 4:** Results of multiple regressions (*p* ≤ 0.05), considering sleep and psychological symptoms during the current period (PSQI, PSQI-A, DASS-Depression, DASS-Anxiety, DASS-Stress scores on T1) as criterion variables and COVID-19-related personal experiences (COVID-19 positivity, forced quarantine, COVID-19-infected relatives/friends, relatives/friends who have died from COVID-19, agreement on government measures) as predictors.

Dependent Variables	Predictors	β	Coefficients of Partial Correlation	t	*p*
PSQI					
R = 0.249 adjusted R^2^ = 0.045 F = 3.707 *p* = 0.003 *	COVID-19 positivity	−0.024	−0.021	−0.356	0.722
	Forced quarantine	−0.006	−0.005	−0.082	0.934
	COVID-19-infected relatives/friends	0.001	0.001	0.017	0.986
	Relatives/friends who have died from COVID-19	0.175	0.173	2.938	0.004 *
	Satisfaction with governmental measures	−0.173	−0.175	−2.974	0.003 *
PSQI-A					
R = 0.307 adjusted R^2^ = 0.078 F = 5.849 *p* = <0.001 **	COVID-19 positivity	−0.015	−0.013	−0.219	0.826
	Forced quarantine	−0.004	−0.003	−0.059	0.953
	COVID-19-infected relatives/friends	−0.038	−0.037	−0.627	0.531
	Relatives/friends who have died from COVID-19	0.229	0.228	3.928	<0.001 **
	Satisfaction with governmental measures	−0.209	−0.213	−3.651	<0.001 **
DASS-Depression					
R = 0.319 adjusted R^2^ = 0.086 F = 6.355 *p* < 0.001 **	COVID-19 positivity	0.013	0.011	0.190	0.849
	Forced quarantine	−0.019	−0.016	−0.273	0.785
	COVID-19-infected relatives/friends	−0.106	−0.105	−1.764	0.079
	Relatives/friends who have died from COVID-19	0.163	0.164	2.794	0.006 *
	Satisfaction with governmental measures	−0.275	−0.276	−4.821	<0.001 **
DASS-Anxiety					
R = 0.360 adjusted R^2^ = 0.114 F = 8.342 *p* < 0.001 **	COVID-19 positivity	−0.016	−0.015	−0.250	0.803
	Forced quarantine	0.021	0.018	0.307	0.759
	COVID-19-infected relatives/friends	−0.065	−0.066	−1.102	0.271
	Relatives/friends who have died from COVID-19	0.226	0.229	3.937	<0.001 **
	Satisfaction with governmental measures	−0.283	−0.288	−5.047	<0.001 **
DASS-Stress					
R = 0.344 adjusted R^2^ = 0.102 F = 7.522 *p* < 0.001 **	COVID-19 positivity	−0.003	−0.003	−0.051	0.959
	Forced quarantine	−0.013	−0.011	−0.185	0.853
	COVID-19-infected relatives/friends	−0.041	−0.041	−0.681	0.497
	Relatives/friends who have died from COVID-19	0.187	0.190	3.249	0.001 *
	Satisfaction with governmental measures	−0.292	−0.295	−5.170	<0.001 **

* Asterisks indicate statistical significance (*p* ≤ 0.05). ** Asterisks indicate statistical significance (*p* < 0.001).

## Data Availability

The data presented in this study are available on request to the corresponding author.

## Supplementary Materials

The following supporting information can be downloaded at: https://www.mdpi.com/article/10.3390/ijerph20021410/s1, Supplementary Materials S1: Checklist for Reporting Results of Internet E-Surveys (CHERRIES) [51]; Supplementary Materials S2: Survey on Google Forms (Italian and English versions).
