# Supplementary material for: Healthcare Workers after Two Years of COVID-19: The Consequences of the Pandemic on Psychological Health and Sleep among Nurses and Physicians
Source: Int J Environ Res Public Health. 2023 Jan 12;20(2):1410. doi: 10.3390/ijerph20021410 (PMC9859438; doi:10.3390/ijerph20021410)
Supplement: Supplementary file 1 [file ijerph-20-01410-s001.zip › ijerph-2130269-supplementary.pdf]

Supplemental material

Supplementary Materials S1: Checklist for Reporting Results of Internet E-Surveys (CHERRIES)

Checklist Item	***Explanation***
Describe survey design	All nurses and medical doctors of the ASL Roma 6 (local health units) were individually invited via mail to participate.
IRB approval	The study has been approved by the local Ethics Committee (Comitato Etico “LAZIO 2”, prot. N. 0223816/2021).
Informed consent	Informed consent was obtained at the beginning of the survey. The form included a brief description of the survey, its questionnaires, its purpose, and approximate time taken to complete it.
Data protection	No personally identifiable information was collected to guarantee anonymity. Identification codes were created to anonymize personal data.
Development and testing	The survey was piloted before the process of data collection, to ensure the usability and technical functionality of the electronic questionnaire.
Open survey versus closed survey	The survey was closed-type and targeted to the healthcare operators of ASL Roma 6.
Contact mode	Investigators sent out the link to the survey by mail, allowing for web-based data entry.
Advertising the survey	This survey was not advertised.
Web/E-mail	This was an e-survey with a link sent out through e-mail to potential participants (all nurses and medical doctors of the ASL Roma 6). Google Forms was used for capturing responses.
Context	The survey was not posted on any web site.
Mandatory/voluntary	The survey was voluntary.
Incentives	No monetary incentives were offered to participants.
Time/Date	The survey was enabled from February 2022 to June 2022 (five months).
Randomization of items or questionnaires	Items were presented in a fixed order.
Adaptive questioning	Adaptive questioning was applied to reduce number and complexity of the questions.
Number of Items	The survey included 91 items.
Number of screens (pages)	The survey included 4 pages:<br>First page: Informed consent<br>Second page: 29 item<br>Third page: 31 item<br>Fourth page: 31 item

Completeness check	The survey was built to have mandatory responses to all questions.
Review step	Respondents were able to review and change their answers through a Back button.
Unique site visitor	N/A
View rate (Ratio of unique survey visitors/unique site visitors)	N/A
Participation rate (Ratio of unique visitors who agreed to participate/unique first survey page visitors)	N/A
Completion rate (Ratio of users who finished the survey/users who agreed to participate)	A total of 287 respondents agreed to participate and completed the survey.
Cookies used	We did not use any cookies.
IP check	We did not use IP address.
Log file analysis	We did not use any log file analysis.
Registration	Identification codes were created at the beginning of the survey. Duplicates were removed (i.e., the first entry was kept).
Handling of incomplete questionnaires	Only completed questionnaires were received and analyzed.
Questionnaires submitted with an atypical timestamp	The timeframe was not used as a cut-off point.
Statistical correction	We did not use weighting of items or propensity scores to adjust for the non-representative sample.

This checklist has been modified from Eysenbach G. Improving the quality of Web surveys: the Checklist for Reporting Results of Internet E-Surveys (CHERRIES). J Med Internet Res. 2004 Sep 29;6(3):e34. doi: 10.2196/jmir.6.3.e34.

Supplementary Materials S2: Survey on Google Forms (Italian and English vesions)

(Italian version)

EMERGENZA COVID-19 E PROFESSIONI SANITARIE: IMPATTO SU SONNO, SALUTE PSICOLOGICA E QUALITÀ DELLA VITA NELLE DIVERSE FASI DELLA PANDEMIA

<u>MODULO DI CONSENSO INFORMATO (prima pagina)</u>

<u>PRIMO BLOCCO (seconda pagina, 29 item)</u>

INFORMAZIONI SOCIO-ANAGRAFICHE

Istruzioni: In questa sezione le verranno richieste alcune informazioni anamnestiche preliminari che non consentiranno in alcun modo di risalire all'identità del singolo individuo. Le ricordiamo che il questionario è interamente anonimo e i dati verranno sfruttati solo per finalità statistiche, pertanto le chiediamo di rispondere con la massima onestà.

- Età___________

- Genere:
  - F
  - M

- Con chi vive attualmente?
  - Da solo
  - Famiglia d'origine
  - Compagno/a o marito/moglie e/o figli
  - Coinquilini/e
  - Altro: ____

- Con quante persone vive attualmente? __________

- Stato civile
  - Celibe/Nubile
  - Coniugato/a
  - Separato/a o Divorziato/a
  - Vedovo/a

- Titolo di studio:
  - Licenza Media
  - Diploma Scuola Superiore
  - Laurea Triennale
  - Laurea Magistrale, Specialistica o Vecchio Ordinamento
  - Dottorato di ricerca

- o Altro: ____________

- Condizione lavorativa attuale
- o Medico
- o Infermiere
- o Altro

- Per favore, indichi gli anni di anzianità di servizio: ________

- Stai attualmente lavorando in prima linea per l'emergenza COVID-19?
- o Sì
- o No

- E' mai risultato positivo al COVID-19 durante l'emergenza sanitaria?
- o Sì
- o No

- E' stata mai predisposta per lei una quarantena obbligatoria?
- o Sì
- o No

- Una persona a lei cara è risultata positiva al COVID-19 durante l'emergenza sanitaria?
- o Sì
- o No

- Ha perso dei cari a causa del COVID-19 durante l'emergenza sanitaria?
- o Sì
- o No

- Ritiene che le misure anti contagio adottate a livello statale siano adeguate?
- o Sì
- o No

SCALA di IMPATTO degli EVENTI

Le seguenti affermazioni si riferiscono all'impatto dell'evento pandemico, con particolare riferimento alla sua fase più acuta. Indichi, per favore, con che frequenza si sono verificate le seguenti condizioni durante la fase acuta di pandemia, apponendo un segno di spunta sulla casella che corrisponde alla sua esperienza. Non ci sono risposte corrette o sbagliate.
Punteggi: scala Likert da 1 ("Mai") a 4 ("Spesso")

1. Ci pensavo anche senza volerlo
2. Evitavo di lasciarmi coinvolgere quando ci pensavo o qualcuno me lo ricordava
3. Cercavo di cancellarlo dalla memoria

4. Avevo difficoltà a dormire o ad addormentarmi perché mi venivano in mente immagini o pensieri sull'accaduto
5. Avevo ondate di emozione sull'accaduto
6. Facevo dei sogni sull'accaduto
7. Mi tenevo alla larga da qualunque cosa potesse ricordarmelo
8. Mi sentivo come se non fosse accaduto o non fosse vero
9. Cercavo di non parlarne
10. Mi venivano in mente all'improvviso delle immagini dell'accaduto
11. Altre cose mi ci facevano pensare
12. Mi rendevo conto di avere ancora molte emozioni sull'accaduto, ma non me ne occupavo
13. Cercavo di non pensarci
14. Qualsiasi cosa collegata mi faceva ritornare in mente le emozioni legate all'accaduto
15. Le mie emozioni sul fatto erano come ovattate

SECONDO BLOCCO

N.B. I seguenti questionari sono stati compilati in riferimento a ciascun periodo della pandemia considerato:

T1: Fase attuale (febbraio-giugno 2022) (SECONDO BLOCCO, parte 1) **(terza pagina, 31 item)**

T0: Fase pre-pandemica (gennaio-febbraio 2020) (SECONDO BLOCCO, parte 2) **(quarta pagina, 31 item)**

PSQI e PSQI-A

1) **A quale ora è solito disposto a letto?**
   Ora __________

2) **Quanto tempo (in minuti) ha impiegato mediamente per addormentarsi ogni sera?**
   Minuti impiegati _____________

3) **A che ora si alza abitualmente?**
   Ora _________

4) **Quante ore di sonno effettivo ha ogni notte? (Le ore di sonno effettivo possono essere diverse dalle ore trascorse a letto)**
   Ore _______________

Per ciascuna delle domande che seguono, scelga la risposta migliore. Risponda per favore a tutte le domande.

5) **Quante volte ha problemi di sonno per ciascuno dei seguenti motivi?**

**a) non riesce ad addormentarsi entro 30 minuti**			
	meno di una volta	una o due volte	tre o più volte
mai ___	alla settimana ____	alla settimana ____	alla settimana ____

b) si sveglia nel mezzo della notte o al mattino presto

	meno di una volta	una o due volte	tre o più volte
mai ___	alla settimana ____	alla settimana ____	alla settimana ____

c) fa uso del bagno

	meno di una volta	una o due volte	tre o più volte
mai ___	alla settimana ____	alla settimana ____	alla settimana ____

d) non riesce a respirare correttamente

	meno di una volta	una o due volte	tre o più volte
mai ___	alla settimana ____	alla settimana ____	alla settimana ____

e) tossisce o russa rumorosamente

	meno di una volta	una o due volte	tre o più volte
mai ___	alla settimana ____	alla settimana ____	alla settimana ____

f) avverte troppo freddo

	meno di una volta	una o due volte	tre o più volte
mai ___	alla settimana ____	alla settimana ____	alla settimana ____

g) avverte troppo caldo

	meno di una volta	una o due volte	tre o più volte
mai ___	alla settimana ____	alla settimana ____	alla settimana ____

h) fa brutti sogni

	meno di una volta	una o due volte	tre o più volte
mai ___	alla settimana ____	alla settimana ____	alla settimana ____

i) ha dei dolori

	meno di una volta	una o due volte	tre o più volte
mai ___	alla settimana ____	alla settimana ____	alla settimana ____

l) per altri motivi. Li descriva per favore ________________________________

Quante volte non riesce a dormire a causa del suddetto motivo

	meno di una volta	una o due volte	tre o più volte
mai ___	alla settimana ____	alla settimana ____	alla settimana ____

6) **Come valuta globalmente la qualità del suo sonno?**
Molto buona___ abbastanza buona ___ abbastanza cattiva___ molto cattiva ____

7) **Quante volte assume farmaci per dormire (prescritti dal medico o comunque ottenuti)?**

	meno di una volta	una o due volte	tre o più volte
mai ___	alla settimana ____	alla settimana ____	alla settimana ____

8) **Quante volte ha difficoltà a stare sveglio durante la guida, durante il pasto o durante un'attività sociale?**

	meno di una volta	una o due volte	tre o più volte
mai ___	alla settimana ____	alla settimana ____	alla settimana ____

9) **In che misura rappresenta per lei un problema avere abbastanza entusiasmo per fare ciò che deve fare?**

nessun problema___	un piccolo problema ____	un certo problema ____	un problema molto grosso ____

10) **Ha problemi mentre dorme poiché**

a) sente vampate di calore:

mai ___	meno di una volta alla settimana ____	una o due volte alla settimana ____	tre o più volte alla settimana ____

b) si sente nervosa:

mai ___	meno di una volta alla settimana ____	una o due volte alla settimana ____	tre o più volte alla settimana ____

c) ha incubi o ricordi di esperienze traumatiche:

mai ___	meno di una volta alla settimana ____	una o due volte alla settimana ____	tre o più volte alla settimana ____

d) ha ansia e panico, non connesso a ricordi traumatici:

mai ___	meno di una volta alla settimana ____	una o due volte alla settimana ____	tre o più volte alla settimana ____

e) fa dei brutti sogni, non connessi a ricordi traumatici:

mai ___	meno di una volta alla settimana ____	una o due volte alla settimana ____	tre o più volte alla settimana ____

f) ha esperienze di terrore o urla durante il sonno senza essere completamente sveglio:

mai ___	meno di una volta alla settimana ____	una o due volte alla settimana ____	tre o più volte alla settimana ____

g) le capita di "agire" i suoi sogni, per esempio gridando, colpendo qualcosa, correndo o scalciando:

mai ___	meno di una volta alla settimana ____	una o due volte alla settimana ____	tre o più volte alla settimana ____

DASS-21

La scala di valutazione è la seguente:
0 = Non mi accade mai
1 = Mi capita qualche volta
2 = Mi capita con una certa frequenza
3 = Mi capita quasi sempre

1 Ho provato molta tensione e ho avuto difficoltà a recuperare uno stato di calma
2 Mi sono accorto di avere la bocca secca

3 Non riuscivo proprio a provare delle emozioni positive
4 Mi sono sentito molto in affanno con difficoltà a respirare (per es. respiro molto accelerato, sensazione di forte affanno in assenza di sforzo fisico)
5 Ho avuto un'estrema difficoltà nel cominciare quello che dovevo fare
6 Ho avuto la tendenza a reagire in maniera eccessiva alle situazioni
7 Ho avuto tremori (per es. alle mani)
8 Ho sentito che stavo impiegando molta energia nervosa
9 Ho temuto di trovarmi in situazioni in cui sarei potuto andare nel panico e rendermi ridicolo
10 Non vedevo nulla di buono nel mio futuro
11 Mi sono sentito stressato
12 Ho avuto difficoltà a rilassarmi
13 Mi sono sentito scoraggiato e depresso
14 Non riuscivo a tollerare per nulla eventi o situazioni che mi impedivano di portare avanti ciò che stavo facendo
15 Ho sentito di essere vicino ad avere un attacco di panico
16 Non c'era nulla che mi dava entusiasmo
17 Sentivo di valere poco come persona
18 Mi sono sentito piuttosto irritabile
19 Ho percepito distintamente il battito del mio cuore senza aver fatto uno sforzo fisico (per es. battito cardiaco accelerato o perdita di un battito)
20 Mi sono sentito spaventato senza ragione
21 Sentivo la vita priva di significato

(English version)

COVID-19 EMERGENCY AND HEALTHCARE PROFESSIONS: IMPACT ON SLEEP, PSYCHOLOGICAL HEALTH AND QUALITY OF LIFE IN THE DIFFERENT PHASES OF THE PANDEMIC

Informed consent (first page)

FIRST BLOCK (second page, 29 items)

SOCIO-DEMOGRAPHIC INFORMATION

Instructions: In this section you will be asked for some preliminary anamnestic information. We remind you that the questionnaire is entirely anonymous and the data will be used only for statistical purposes, therefore we ask you to respond with the utmost honesty.

- Age___________

- Gender:
  - o F
  - o M

- Who do you currently live with?
  - o Alone
  - o Parents
  - o Partner or husband/wife and/or children
  - o Roommates
  - o other: ____

- How many people do you currently live with? __________

- Marital status
  - o Single
  - o Married
  - o Separated o Divorced
  - o Widower

- Qualification:
  - o Middle License
  - o High School Diploma
  - o Bachelor's degree
  - o Master's Degree
  - o PhD
  - o other: ____

- Current working condition
- o Medical doctor
- o Nurse
- o other

- Please indicate the years of service: ________

- Are you currently working on the front lines of the COVID-19 emergency?
- o Yes
- o No

- Have you ever tested positive for COVID-19 during the health emergency?
- o Yes
- o No

- Have you ever been subjected to a forced quarantine period?
- Yes
- o No

- Have relatives or friends tested positive for COVID-19 during the health emergency??
- o Yes
- o No

- Did relatives or friends die from COVID-19 during the health emergency?
- o Yes
- o No

- Do you think that the national anti-contagion measures are adequate?
- o Yes
- o No

IMPACT OF EVENT SCALE

The following statements refer to the impact of the pandemic event, with particular reference to its most acute phase. Please indicate how often the following conditions occurred during the acute phase of the pandemic by ticking the box that corresponds to your experience. There are no right or wrong answers.
Scores: Likert scale from 1 (“Never”) to 4 (“Often”).

1. I thought about it even when I did not mean to
2. I avoided letting myself get upset when
3. I tried to remove it from my memory

4. I had trouble falling asleep or staying asleep because pictures or thoughts about it came into my mind
5. I had waves of strong feelings about it
6. I had dreams about it
7. I stayed away from reminders of it
8. I felt as if it hadn’t happened or it wasn’t real
9. I tried not to talk about it
10. Pictures about it popped into my mind
11. Other things kept making me think about it
12. I was aware that I still had a lot of feelings about it, but I didn’t deal with them
13. I tried not to think about it
14. Any reminder brought back feelings about it
15. My feelings about it were kind of numb

SECOND BLOCK

N.B. The following questionnaires were completed with reference to a specific period of the pandemic:
T1: Current phase (February-June 2022) (SECOND BLOCK, part 1) (**third page, 31 items**)
T0: Pre-pandemic period (January-February 2020) (SECOND BLOCK, part 2) (**fourth page, 31 items**)

PSQI and PSQI-A

1) **When have you usually gone to bed at night?**
   USAL BED TIME __________

2) **How long (in minutes) has it usually take you to fall asleep each night?**
   MINUTES _____________

3) **When have you usually gotten up in the morning??**
   USUAL GETTING UP TIME _________

4) **How many hours of actual sleep did you get at night? (This may be different than the number of hours you spend in bed.)**
   HOURS OF SLEEP PER NIGHT _______________

For each of the remaining questions, check the one best response. Please answer all questions.

5) **How often have you had trouble sleeping because you...**

a) Cannot get to sleep within 30 minutes

Never __	Less than once a week ____	Once or twice a week ____	Three or more times a week ____

b) Wake up in the middle of the night or early morning

	Less than once a	Once or twice a	Three or more times

Never __ | Less than once a week ____ | Once or twice a week ____ | Three or more times a week ____

c) Have to get up to use the bathroom

Never __ | Less than once a week ____ | Once or twice a week ____ | Three or more times a week ____

d) Cannot breathe comfortably

Never __ | Less than once a week ____ | Once or twice a week ____ | Three or more times a week ____

e) Cough or snore loudly

Never __ | Less than once a week ____ | Once or twice a week ____ | Three or more times a week ____

f) Feel too cold

Never __ | Less than once a week ____ | Once or twice a week ____ | Three or more times a week ____

g) Feel too hot

Never __ | Less than once a week ____ | Once or twice a week ____ | Three or more times a week ____

h) Had bad dreams

Never __ | Less than once a week ____ | Once or twice a week ____ | Three or more times a week ____

i) Have pain

Never __ | Less than once a week ____ | Once or twice a week ____ | Three or more times a week ____

j) Other reason(s), please describe ______________________________

How often during the past month have you had trouble sleeping because of this?

Never __ | Less than once a week ____ | Once or twice a week ____ | Three or more times a week ____

6) **How would you rate your sleep quality overall?**

Very good___ Fairly good ___ Fairly bad ___ Very bad ____

7) **How often have you taken medicine (prescribed or "over the counter") to help you sleep?**

Never _ | Less than once a week ____ | Once or twice a week ____ | Three or more times a week ____

8) **How often have you had trouble staying awake while driving, eating meals, or engaging in social activity?**

Never _ | Less than once a week ____ | Once or twice a week ____ | Three or more times a week ____

9) **How much of a problem has it been for you to keep up enough enthusiasm to get things done?**

No problem at | Only a very slight | Somewhat of a | A very big problem

all__ problem ____ problem ____ ____

10) How often have you had trouble sleeping because you…

a) Feel hot flashes:

Never __	Less than once a week ____	Once or twice a week ____	Three or more times a week ____

b) Feel general nervousness:

Never __	Less than once a week ____	Once or twice a week ____	Three or more times a week ____

c) Had memories or nightmares of a traumatic experience:

Never __	Less than once a week ____	Once or twice a week ____	Three or more times a week ____

d) Had severe anxiety or panic, not related to traumatic memories:

Never __	Less than once a week ____	Once or twice a week ____	Three or more times a week ____

e) Had bad dreams, not related to traumatic memories:

Never __	Less than once a week ____	Once or twice a week ____	Three or more times a week ____

f) Had episodes of terror or screaming during sleep without fully awakening:

Never __	Less than once a week ____	Once or twice a week ____	Three or more times a week ____

g) Had episodes of "acting out" your dreams, such as kicking, punching, running, or screaming:

Never __	Less than once a week ____	Once or twice a week ____	Three or more times a week ____

DASS-21

The rating scale is as follows:
0 = Did not apply to me at all
1 = Applied to me to some degree, or some of the time
2 = Applied to me to a considerable degree or a good part of time
3 = Applied to me very much or most of the time

1 I found it hard to wind down
2 I was aware of dryness of my mouth
3 I couldn’t seem to experience any positive feeling at all
4 I experienced breathing difficulty (e.g. excessively rapid breathing, breathlessness in the absence of physical exertion)

5 I found it difficult to work up the initiative to do things
6 I tended to over-react to situations
7 I experienced trembling (e.g. in the hands)
8 I felt that I was using a lot of nervous energy
9 I was worried about situations in which I might panic and make a fool of myself
10 I felt that I had nothing to look forward to
11 I found myself getting agitated
12 I found it difficult to relax
13 I felt down-hearted and blue
14 I was intolerant of anything that kept me from getting on with what I was doing
15 I felt I was close to panic
16 I was unable to become enthusiastic about anything
17 I felt I wasn’t worth much as a person
18 I felt that I was rather touchy
19 I was aware of the action of my heart in the absence of physical exertion (e.g. sense of heart rate increase, heart missing a beat)
20 I felt scared without any good reason
21 I felt that life was meaningless
